# Protective Effect of Royal Jelly against Cyclophosphamide-Induced Thrombocytopenia and Spleen and Bone Marrow Damages in Rats 

**DOI:** 10.22074/cellj.2020.6703

**Published:** 2019-12-15

**Authors:** Fatemeh Khazaei, Elham Ghanbari, Mozafar Khazaei

**Affiliations:** 1 Student Research Committee, Kermanshah University of Medical Sciences, Kermanshah, Iran; 2 Fertility and Infertility Research Center, Health Technology Institute, Kermanshah University of Medical Sciences, Kermanshah, Iran

**Keywords:** Bone Marrow, Cyclophosphamide, Platelet, Spleen, Thrombocytopenia

## Abstract

**Objective:**

Despite the effective role of chemotherapy in cancer treatment, several side effects have been reported to date. For instance, Cyclophosphamide (CP) induces deleterious effects on both cancer and normal cells. Royal jelly (RJ) has a lot of beneficial properties, such as anti-oxidant and anti-inflammatory activities. The aim of the present study was to examine the protective effect of RJ against CP-induced thrombocytopenia, as well as bone marrow, spleen, and testicular damages in rats.

**Materials and Methods:**

In this experimental study, 48 male Wistar rats were divided into six groups (n=8/group); control, CP, RJ (100 mg/kg), RJ (200 mg/kg), RJ (100 mg/kg)+CP, and RJ (200 mg/kg)+CP groups. RJ was administered orally for 14 days. Then, CP at concentrations of 100, 50, and 50 mg/kg was intraperitoneally injected at day 15, 16, 17, respectively. The animals were sacrificed three days after the last injection of CP. Hematological parameters, serum levels of platelet factor 4 (PF4), nitric oxide (NO), and ferric reducing antioxidant power (FRAP) were measured. Also, the pathological analysis of bone marrow, spleen, and testicles was assessed.

**Results:**

CP caused a significant decrease in the number of platelets, white and red blood cells (P<0.001), as well as the levels of FRAP (P<0.01), whereas the serum levels of PF4 and NO were significantly increased. These detrimental alterations were significantly reversed to the baseline upon pretreatment of rats with RJ in the RJ100+CP and RJ200+CP groups (P<0.05). CP caused histological changes in bone marrow, spleen, and testes. Pretreatment with RJ showed noticeable protection against these harmful effects.

**Conclusion:**

RJ prevented CP-induced biochemical and histological damages.

## Introduction

Cyclophosphamide (CP) is a chemotherapeutic alkylating agent widely used against a variety of malignant tumors and some immune diseases. Also, it also been used as an immunosuppressive agent for organ transplantation, multiple sclerosis, and systemic lupus erythematosus (1). Like other chemotherapeutic drugs, CP has a broad range of side effects such as the reduction in the number of platelets (PLTs), white and red blood cells (WBCs, RBCs). It can cause severe thrombocytopenia, as well (2).

Thrombocytopenia, defined as a decrease in the number of PLTs to less than 150,000/mL, is a common side effect of chemotherapy and one of the lethal hematological disorders (3). Its occurrence is either due to inhibited/ insufficient production of PLT in bone marrow or increased destruction of the cells (in malaria and dengue fever). In this context, most of the chemotherapeutic agents can result in the development of thrombocytopenia (4). PLT factor 4 (PF4) is an important mediator in blood coagulation, released from alpha-granules of the activated PLTs. It plays a significant role in blood coagulation, wound healing (5), and inflammation (6). The blood usually contains very low amounts of PF4, and only in pathological conditions, such as sepsis and acute tissue injury, high levels of PF4 release from the activated PLTs into blood (7).

Histological evaluation of the bone marrow in thrombocytopenic patients indicated a marked rise in the number of megakaryocytes, implying that the disorder is mainly caused by the destruction of peripheral PLT without a suitable bone marrow compensation (8). Also, morphological alterations are usually detected in the spleen of patients after the injection of CP, which include the depletion of white and red cells. Also, the bone marrow showed hematopoietic cells reduction (9). As shown in previous studies, the counts of splenic and bone marrow cells are decreased in cyclophosphamidetreated mice due to oxidative stress (OS) caused by the metabolite compounds of CP (10). It is well-known that chemotherapeutic drugs induce thrombocytopenia by two primary mechanisms: an increase in PLT destruction or a decrease in PLT production by apoptosis of megakaryocytes (11).

Also, CP has cytotoxic effects on rapidly proliferating tissues such as testicles which are more sensitive to its toxic impacts. Following therapy of cancer with CP, oligo- and azoospermia lead to male infertility (12, 13). Moreover, experimental studies have also shown that treatment of mice or rats with CP resulted in decreased sperm counts and sperm motility, as well as the reduced testosterone concentrations (14, 15). On the other hand, CP not only influences cancer cells but also affects normal cells, and it can increase the formation of reactive oxygen species (ROS) and nitric oxide (NO), leading to peroxynitrite generation which damages the cellular proteins, DNA, and lipids (16). It seems that antioxidant compounds should be able to inhibit the harmful effects of ROS during the use of chemotherapy drugs (17).

Royal jelly (RJ) has different medicinal properties, including antioxidant and anti-inflammatory potential, as well as enhancement of immune activity and infertility improvement (18, 19). The antioxidant activity and scavenging potency of RJ were reported against free radicals such as superoxide anions, hydroxyl, and DPPH (1, 1 diphenyl-2-picrylhydrazyl) radicals in several studies. Also, the beneficial effects of RJ supplementation on the reproductive system have been addressed in different animals (19).

Chemotherapy induced-thrombocytopenia is a major clinical problem in cancer therapy. However, no appropriate treatment and/or preventive strategy to resolve this problem. Hence, there is a need for new factors that would be enabled to protect normal cells and tissues against chemotherapy-induced toxicity with no protection against tumor cells. It seems that the combination of the drug with an antioxidant agent can be an appropriate approach to decrease the side effects of CP (20). The aim of this study was to investigate the protective effect of RJ pretreatment against thrombocytopenia, oxidative stress, as well as bone marrow, spleen, and testicular damages induced by CP in rats.

## Materials and Methods

In this experimental study, male Wistar rats (200 ± 20 g) were kept under standard laboratory conditions at the temperature of 24˚C, the relative humidity of 60-70%, and a 12/12-hour light/dark cycle. All animals had free access to standard chow and tap water. This experimental study was carried out in accordance with the guide for the care and use of laboratory animals and approved by the Local Ethics Committee of Kermanshah University of Medical Sciences with a code number IR.KUMS.REC.1397. 296.

The fresh RJ was provided from local beekeeping (Urmia, Iran), and was stored until the use in a freezer. Also, the quality of RJ was approved by an expert academic member of the Urmia University of Medical Sciences. The CP (Baxter Oncology, Germany Lot No.7E074A) was provided by national Co. (Iran).

### Study protocol

Rats were divided into six groups (n=8/group): 1) Control group was orally administered 0.5 ml distilled water (RJ solvent) for 2 weeks. 2) CP group was orally received 0.5 ml distilled water for 14 days, and then CP was injected intraperitoneally (IP) at doses of 100, 50 and 50 mg/kg at days 15, 16, and 17, respectively (21). 3, 4) RJ groups orally received 100 or 200 mg/ kg/day RJ for 14 days. 5, 6) RJ+CP groups were orally received 100 or 200 mg/kg/day RJ for 14 days. The doses of RJ were selected based on our pervious study conducted on rats (22). Afterward, CP at concentrations of 100, 50, and 50 mg/kg was administered at days 15, 16, and 17, respectively.

The body weight of rats was measured on day 1, and the day when the study was finished. After 72 hours of the last CP injection, rats were sacrificed after an overnight fast. Blood samples were collected from the heart and divided into two parts; the first part was collected in anticoagulant tubes for blood analysis. Then, sera were isolated from the second part of the blood samples and used for the measurement of PF4, NO, and FRAP levels. Conversely, spleen, femur-derived bone marrow tissue, and testes were removed immediately and fixed in formalin (10%). The weight of spleen were determined, and it ratios to body weight were calculated using the following formulas: [weight of the spleen (g)/body weight of the rat (g)] × 100 (23).

### Blood analysis

The blood was collected into tubes containing EDTA as an anticoagulant agent to determine PLTs, WBCs, and RBCs counts using an automated hematology analyzer (Sysmex XW™-100, America).

### PF4 measurement

Serum level of PF4 was analyzed using the PF4 ELISA kit according to the manufacturer’s instructions. Ultimately, the absorbance was measured at 450 nm with an ELISA reader (Stat fax 100, USA).

### FRAP assay

The reduction of Fe^+3^ to Fe^+2^ by antioxidant compounds was monitored (22). The working FRAP solution was prepared by mixing 1 ml of 2,4,6-tripyridyl-s-triazine (40 mM dissolved in 40 mM HCl) and 1 ml of FeCl3.6H2O (20 mM in water) with 10 ml of acetate buffer (300 mM, pH=3.6). Next, the mixture was heated to 37˚C for 10 minutes before the use. For a manual FRAP assay, 200 μl of serum samples were added to 1.5 ml of working FRAP solution. The mixtures were incubated in the dark at 37˚C for 30 minutes, and then the absorbance of samples was recorded at 593 nm by a spectrophotometer device.

### Nitric oxide assay

The serum levels of NO were determined according to the Griess method (24). Briefly, 400 μL of serum samples were deproteinized by adding 6 mg of zinc sulfate and then centrifuged (12 minutes, 12000 g/4˚C). Standard solutions were prepared as 0, 6.25, 12.5, 25, 50, 100, and 200 μM nitrite. Afterward, 100 μL of deproteinized samples were poured into wells, and 100 μL of vanadium chloride was added to all wells, followed by rapid addition of 50 μL of sulfanilamide, and 50 μL of N-(1-naphthyl) ethylene diamine di-hydrochloride. The mixture was incubated for 30 minutes, and then the absorbance was measured at the wavelengths of 450 and 630 nm using an ELISA Reader (Statfax 100, USA).

### Histopathological analysis

The spleen, femoral bone marrow, and testes were slowly rinsed with phosphate-buffered saline (PBS), dried, weighed, and consequently fixed in 10% formalin. The tissues were dehydrated using the ascending grades of ethanol, then cleared in xylene, and finally embedded in paraffin wax. The tissue sections (5 μm) were prepared and dried at 37˚C in an incubator. The sections were deparaffinized in xylene and rehydrated by the descending grades of ethanol and stained with hematoxylin and eosin. The slides were evaluated for histological analysis under a light microscope (×10 and ×40 magnification). The images were captured by a calibrated microscope connected to a computer equipped with the KECAM software.

For the spleen histological analysis, the following parameters were used: the diameter and count of white pulps per sections, hemosiderin deposition, as well as red and white pulp cellularity. Also, cellularity of femoral bone marrow, including the number of megakaryocytes, was determined. The histological changes in testicular tissues, including seminiferous tubule diameters (STD) and atrophy, were measured (25).

### Statistical analysis

All data were expressed as the mean ± SE and analyzed using the SPSS software package version 18 (Inc. Chicago, IL, USA). The difference among the groups was also analyzed by one-way analysis of variance (ANOVA), followed by Duncan post hoc test. The P<0.05 were considered statistically significant.

## Results

**Fig 1:**
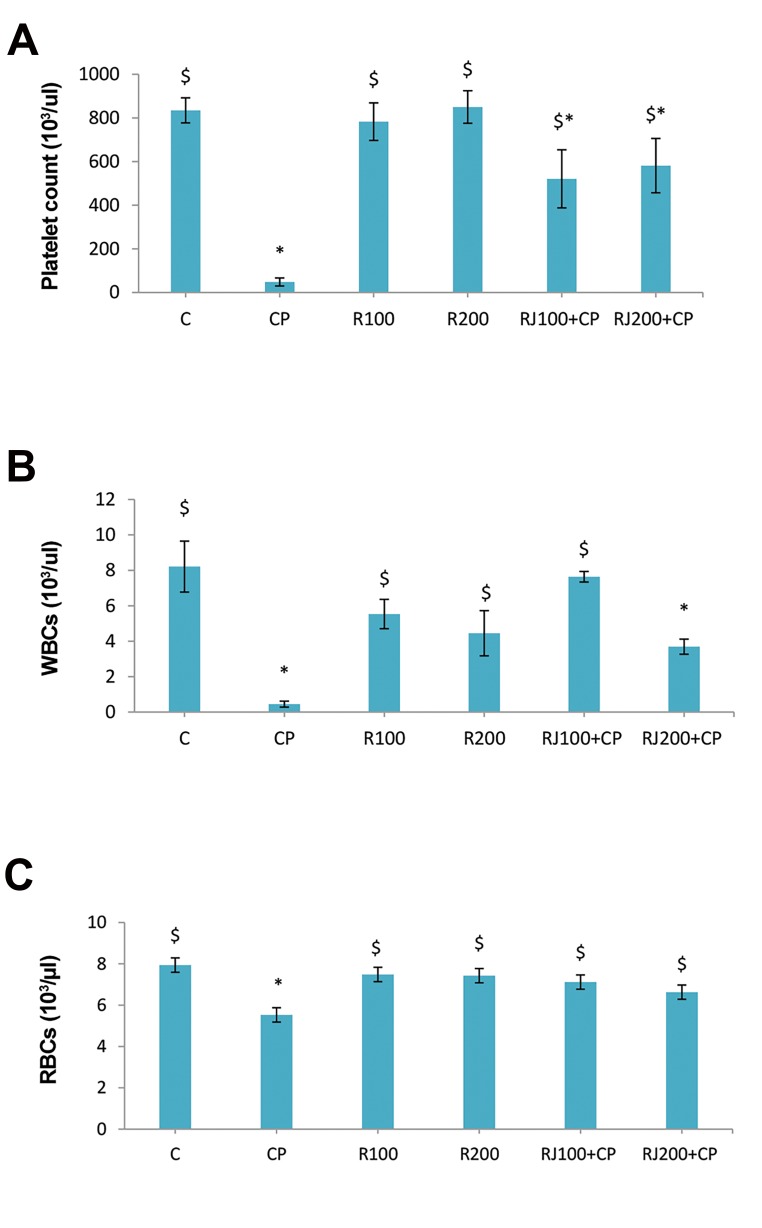
Changes of blood cells in different groups. RJ pretreatment and CPinduced changes in the number of **A.** PLT, **B.** WBCs, and **C.** RBCs. Data are represented as the mean ± SE (n=8). *; Significant (P<0.05) difference vs. the control group, $; Significant (P<0.05) difference versus the CP group, C; Control, CP; Cyclophosphamide, R; Royal Jelly, PLT; Platelets, WBC; White blood cell, and RBC; Red blood cell.

The administration of CP in rats led to a significant (P<0.001) decrease in the number of PLTs (48.11 ± 18.35×10^3^/µl) and caused severe thrombocytopenia. The pretreatment of rats with RJ (100 and 200 mg/kg) increased the number of PLTs in the RJ+CP groups in a dose-dependent manner (Fig .1A). Also, the administration of CP significantly (P<0.001) decreased the frequency of WBCs (0.45 ± 0.17×10^3^/µl), while RJ (100 and 200 mg/kg) increased the number of WBCs in the RJ+CP groups, and the highest increase was observed in the RJ100+CP group which was the same as the control group (Fig .1B). The administration of CP significantly (P=0.001) diminished the number of RBCs (5.528 ± 0.46×10^6^/µl), but the reduction was not as great as that of observed in PLTs and WBCs. The administration of RJ increased the number of RBCs in the RJ+CP groups; however, no significant difference was shown when compared with the control group (Fig .1C).

### PF4 levels

**Fig 2:**
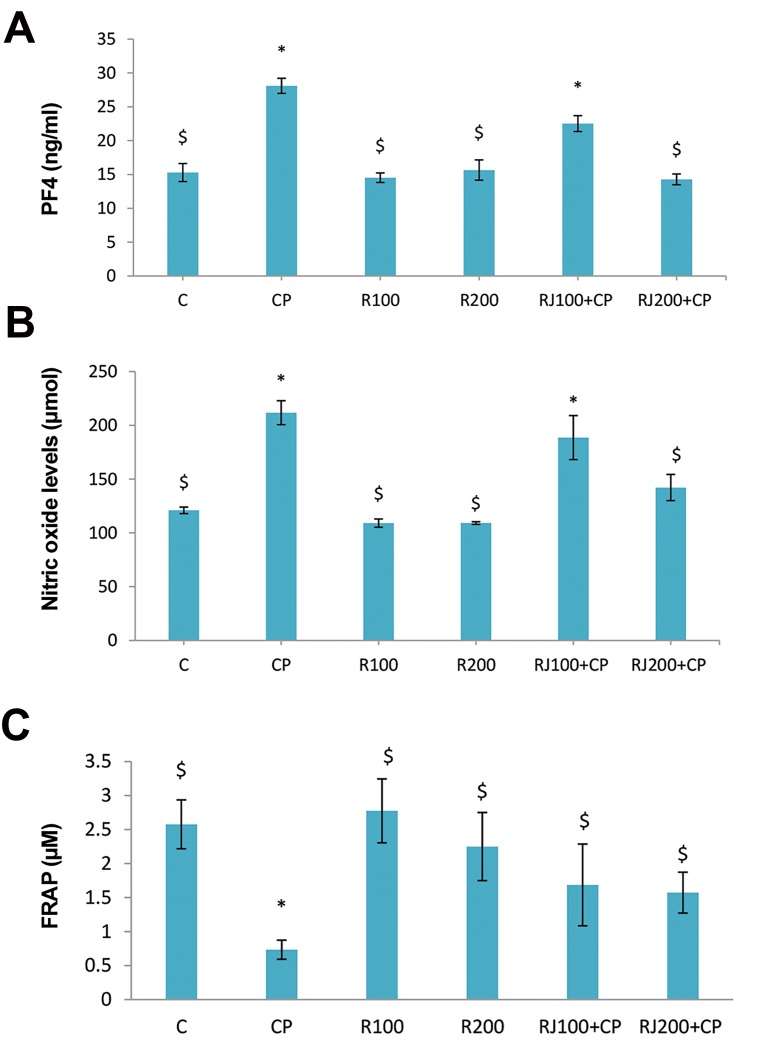
Changes of biochemical factors of serum. The effect of RJ on CP-induced changes of **A.** PF4 level, **B.** NO, and **C.** FRAP in serum samples of study groups. Values are expressed as the mean ± SE (n=8). *; Significant (P<0.05) difference versus the control group, $; Significant (P<0.05) difference versus the CP group, C; Control, CP; Cyclophosphamide, R; Royal Jelly, PF4; Platelets Factor 4, FRAP; Ferric reducing antioxidant power, and NO; Nitric oxide.

CP significantly (P<0.001) increased the serum level of PF4 (28.10 ± 1.11 vs. 15.29 ± 4.91 ng/ml). RJ alone caused no change in the concentration of PF4, while it decreased PF4 levels in the RJ+CP groups in a dose-dependent manner and reached the level of PF4 to the normal level as observed in the RJ200+CP group (Fig .2A).

### Nitric oxide, and FRAP levels

CP significantly (P<0.001) increased NO levels. The level of NO was decreased in the RJ+CP groups in a dose-dependent manner, but RJ alone did not change the concentration of NO (Fig .2B).

The serum levels of FRAP showed a significant decrease in the CP group (P<0.01, Fig .2C). There was a significant increase in FRAP levels in the RJ+CP groups compared to the CP group. RJ (100 and 200) elevated the levels of FRAP when compared to the control group; however, the increase was not statistically significant.

### Body weight

**Fig 3:**
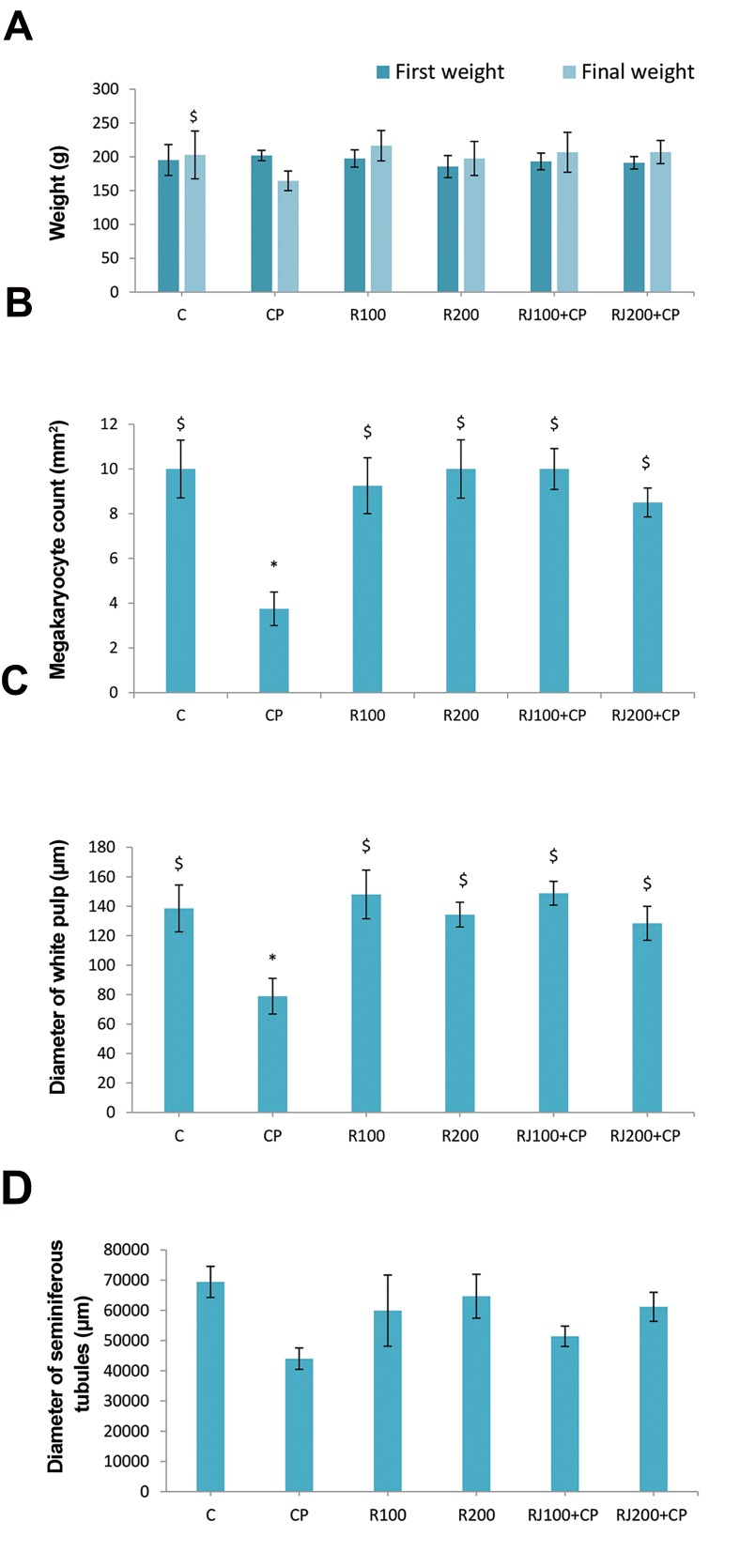
The impact of CP and RJ on the body weight, bone marrow, spleen, and testes in rats.** A.** Initial and final BW, **B.** Count of megakaryocytes, **C.** Diameter of white pulps, and D. Diameter of seminiferous tubules in the control and experimental groups. Data are presented as the mean ± SE (n=8). *; Significant (P< 0.05) difference versus the control group, $; Significant (P<0.05) difference versus the CP group, C; Control, CP; Cyclophosphamide, and R; Royal Jelly.

CP significantly (P=0.001) decreased BW (164.4 ± 14.5 vs. initial weight 201.9 ± 7.6). RJ treatment increas BW in all groups, and there was no significant difference between RJ groups compared with the control group. RJ protects BW loss in the RJ+CP groups. RJ alone did not change BW, but it increased BW in the RJ+CP groups; however, the increase was not statistically meaningful (Fig .3A). There was a significant increase (P<0.01) in the spleen/BW ratio in the CP group (0.59 ± 0.05 vs. control 0.35 ± 0.01), however, it was normalized in the RJ+CP groups (0.33 ± 0.01 and 0.45 ± 0.06), and no significant difference was found when compared with the RJ groups.

### Histological changes

**Fig 4:**
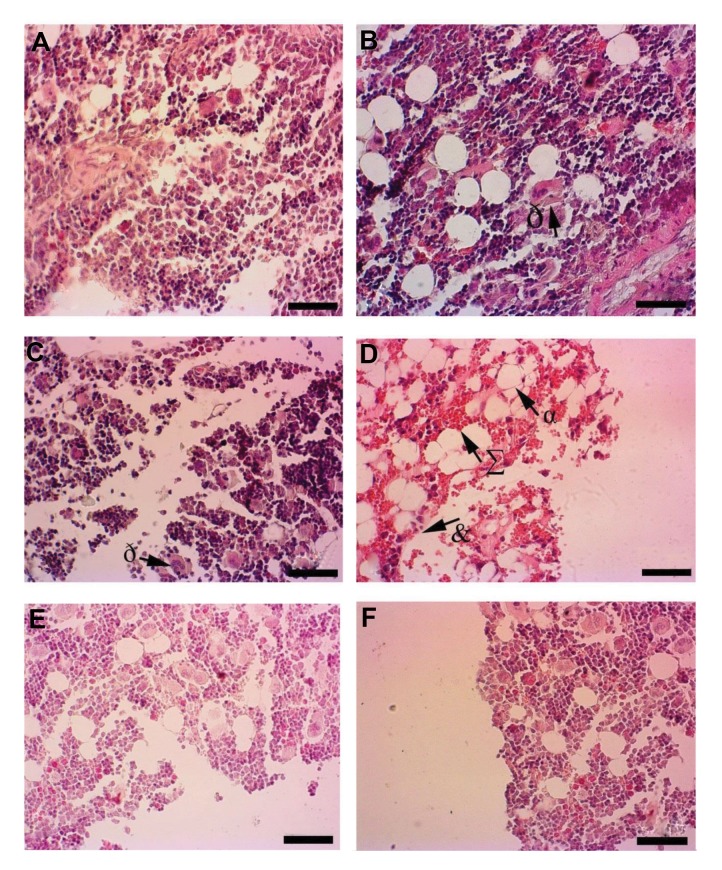
Hisopathological changes of femoral bone marrow in different groups. **A.** The Control, **B.** R100, **C.** R200 groups, showing no pathological changes, megakaryocytes (ð). **D.** The CP group with decreased cellularity in hematopoietic cells (&), severe hemorrhage (∑) and adipose-like cells (α), **E.** CP+100 group, and **F.** CP+200 group (H&E, ×40) (scale bar: 100 µm). CP; Cyclophosphamide and R; Royal Jelly.

Some histological changes of bone marrow (number of megakaryocyte), spleen (white pulp), and testes (seminiferous tubules) were shown in (Fig .3B-D). In control groups, bone marrow showed normal histology (Fig .4A). CP decreased the number of hematopoietic cells in the bone marrow and showed severe hemorrhage and an increase in the frequency of adipose-like cells (Fig .4B). RJ alone showed no pathological changes in bone marrow (Fig .4C, D). RJ+CP-treated rats protected bone marrow against CP tissue injuries (Fig .4E, F).

The number of megakaryocytes was significantly lower in the CP group compared with other groups, while it was considerably higher in the RJ+CP groups in comparison with other experimental groups (P<0.01, Fig .3B).

**Fig 5:**
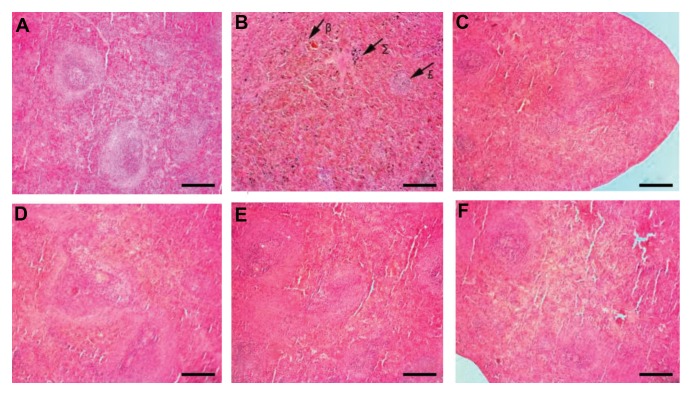
The spleen sections (H&E, ×10). **A.** The control group shows the normal architecture of spleen. **B.** The CP group indicates a decrease in the diameter of white pulps (£), the increase rate of hemorrhage (β), hemosiderin deposition (∑), as well as the red and white pulp cellularity. **C.** The R100, **D.** R200 groups demonstrate the normal structure, E. The CP+100, and F. CP+200 groups show the decreased hemosiderin deposition (scale bar: 50 µm). CP; Cyclophosphamide and R; Royal Jelly.

Splenic histology didn’t showed changes in control group (Fig .5A), CP led to disorganization in splenic structures such as hemosiderin deposition and reduction of the diameter of white pulp (Fig .5B). RJ alone did not affect the structure of spleen (Fig .5C, D) and it was similar to the control group. White pulp diameter in the CP group was significantly decreased; whereas, it was increased in the RJ+CP groups (P<0.01, Fig .3C). RJ+CP decreased hemosiderin deposition (Fig .5E, F).

**Fig 6:**
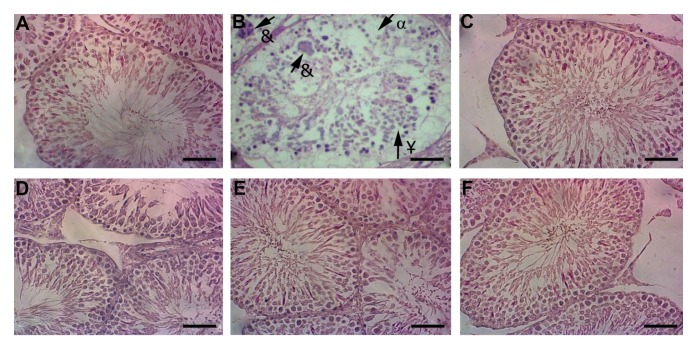
The testicular sections (H&E, ×40). **A.** The control group, **B.** The CP group indicates the severe degenerative changes in seminiferous tubules (¥), decreased number of germ cells (α) and presence of giant multinucleated cells (&). **C.** The R100, **D.** R200 groups were the same as the control group. **E.** The CP+100, and F. CP+200 groups show the morphological structure of the seminiferous tubules as the same as the control group (scale bar: 100 µm). CP; Cyclophosphamide and R; Royal Jelly.

Testicular histology of the control group didn’t showed changes (Fig .6A). Severe degenerative alterations were found in the CP group, characterized by a decreased number of germ cells (seminal linage) with disorganized morphology in seminiferous tubules, as well as the presence of multinucleated giant cells (Fig .6B). Cellular arrangement in seminiferous tubules was the same as the RJ and control groups (Fig.6C, D). RJ protects testicular tissues against CP toxicity in the RJ+CP groups (Fig .6E, F). The diameter of seminiferous tubules in the CP group was decreased; however, it was not statistically significant. Also, there was no significant difference when compared with other groups (Fig .3D).

## Discussion

In this experimental study, RJ pretreatment protected the animals against the side effects of CP injection on the cell number of PLTs and WBCs, levels of serum biochemical factors, and histological structures of bone marrow, spleen, and testes. The main aim of this study was to find anti-thrombocytopenic properties of RJ. To our knowledge, this is the first report on the protective effect of RJ against CP-induced thrombocytopenia and some other side effects. Also, RJ normalizes serum levels of PF4, NO, and FRAP

The adverse effects of CP in the therapy of solid tumors, lymphomas, and leukemia are well characterized such as bone marrow suppression, leading to the reduction of PLTs and WBCs, the impairment of organ functions in patients, and the reduction of the quality of life in patients (1, 26). CP-induced thrombocytopenia and leucopenia (27) can increase the PLT destruction and/or reduce the PLTs production in bone marrow (28). It is mainly associated with bleeding and prolonged clotting due to the lowered number of PLTs (29).

The present model of animal thrombocytopenia in rats was introduced by our previous study (21). After CP injection, similar clinical symptoms of thrombocytopenia such as anorexia, diarrhea, weight loss, and alopecia were observed. RJ pretreatment protects thrombocytopenia and leucopenia. These data were in agreement with a previous study, which showed that CP-induced leucopenia in mice (30).

The antioxidant compounds had beneficial effects on the treatment course of patients with immune thrombocytopenia (31). On the other hand, it was documented that the use of antioxidant supplementation protects CP-induced toxicity (32). The protective role of RJ against CP-induced OS could be attributed to its antioxidant properties. It should be noted that the animals in the RJ+CP groups received RJ (14 days) before CP that was injected at days 15, 16, and 17, and no direct interference was evident when RJ and CP were applied. RJ has many components, such as growth factors and immune modulator compounds (19), which can protect bone marrow and other organs against CP toxicity.

PF4 decreased the production of PLTs through the inhibition of colony growth *in vivo* (33). We showed that CP increased the serum levels of PF4 in thrombocytopenic rats. Pretreatment of rats with RJ (100 and 200 mg/kg) dramatically reversed the detrimental effects caused by the administration of CP. RJ has been shown to have strong antioxidant properties, protecting organs, tissues, and cells against oxidative injuries caused by free radicals (19).

CP treatment increased the serum level of NO (one of the indices of oxidative stress) in rats, suggesting that CP can cause oxidative damage. Also, the serum levels of FRAP were lower in the CP group compared with treatment groups. RJ pretreatment increased FRAP levels and decreased the serum levels of NO, indicating that RJ prevented CP-induced elevation of NO and reduction of FRAP.

The count of nucleated cells in the bone marrow is a direct index of the process of hematopoiesis. A reduction in the number of these cells in the CP group showed the acute injuries in bone marrow and apoptosis of these cells, although this damage was not apparent in rats treated with RJ+CP. The spleen can perform compensatory hematopoiesis and restore this hematological process when the bone marrow function is disrupted (34). Consistent with previous studies, we showed that the injection of CP decreased the number of megakaryocytes in the bone marrow (11). However, RJ pretreatment exhibited that megakaryocyte was significantly increased in rats. So, we concluded that RJ pretreatment might mitigate thrombocytopenia through the alleviation of the loss of bone marrow cells in CP-induced cytotoxicity.

The splenic histology changed due to oxidative stress, following the administration of CP. These alterations were significantly improved in the RJ+CP groups, which can be attributed to RJ pretreatment, abrogating CP-induced spleen atrophy. Also, the increase of the diameter of white pulps indicates that RJ pretreatment can promote the recovery of this damage after CP administration.

Patients with cancer have low-antioxidant capacity before initiating therapy; therefore, chemotherapeutic compounds exacerbate OS as indicated by lipid peroxidation and DNA oxidation after and/or during cancer treatment. Natural antioxidants before or after the administration of these agents protect normal cells from additional OS and treatment-induced toxicity (35).

In this study, disorganization in the seminiferous tubules and presence of giant multinucleated cells, and decreased diameter of seminiferous tubules were observed in rats after CP treatment that the results were in agreement with the findings of the previous study (36). RJ pretreatment in the RJ+CP groups reversed these alterations. In line with this finding, our previous study showed that RJ ameliorated diabetes-induced impairment in the testicular tissue, probably caused by its antioxidant activity (25).

## Conclusion

The current evidence increases the possibility of RJ potential to normalize the number of PLTs in thrombocytopenic rats caused by chemotherapy. We showed that RJ protected CP-induced thrombocytopenia, as well as the changes in other hematological parameters, probably as a result of its antioxidant and anti-cancer properties. CP-induced histopathological changes in organs were prevented by RJ pretreatment. Thus, RJ can be suggested as a food supplement to ameliorate the adverse effects of chemotherapeutic drugs.
